# Regulatory T cells in cancer: from immunosuppression to therapeutic targeting

**DOI:** 10.3389/fimmu.2025.1703211

**Published:** 2025-11-27

**Authors:** Paola Basurto-Olvera, Hector Serrano, Carmen Maldonado-Bernal

**Affiliations:** 1 Unidad de Investigación en Inmunología y Proteómica, Hospital Infantil de México Federico Gómez, Mexico City, Mexico; 2 Posgrado en Biología Experimental, Universidad Autónoma Metropolitana, Unidad Iztapalapa, Mexico City, Mexico; 3 Laboratorio de Biología Molecular y Regulación Endocrina, Universidad Autónoma Metropolitana, Unidad Iztapalapa, Mexico City, Mexico

**Keywords:** Tregs cells, immunotherapy, cancer, leukemia, suppressor cells, immune checkpoints, cellular plasticity, Treg depletion

## Abstract

Regulatory T cells (Tregs) play a pivotal role in maintaining immune homeostasis; however, their presence in the tumor microenvironment contributes to immune evasion and cancer progression. The modulation of Tregs has emerged as a key strategy in immunotherapy, with approaches ranging from direct depletion to functional reprogramming. This review summarizes advances in Treg modulation through checkpoint blockade, selective depletion, and metabolic or epigenetic reprogramming. Additionally, we discuss the potential of Treg plasticity as a therapeutic avenue, emphasizing how shifts in Treg phenotype can enhance antitumor immunity. Furthermore, we highlight combinatory strategies, including radiotherapy, cytokine-based therapies, and metabolic targeting that reshape the immune landscape to potentiate cancer immunotherapy. Understanding the dynamic nature of Tregs cells and their modulation offers promising directions for enhancing therapeutic efficacy and overcoming resistance in several cancer types.

## 1 Introduction

**Figure 1:**
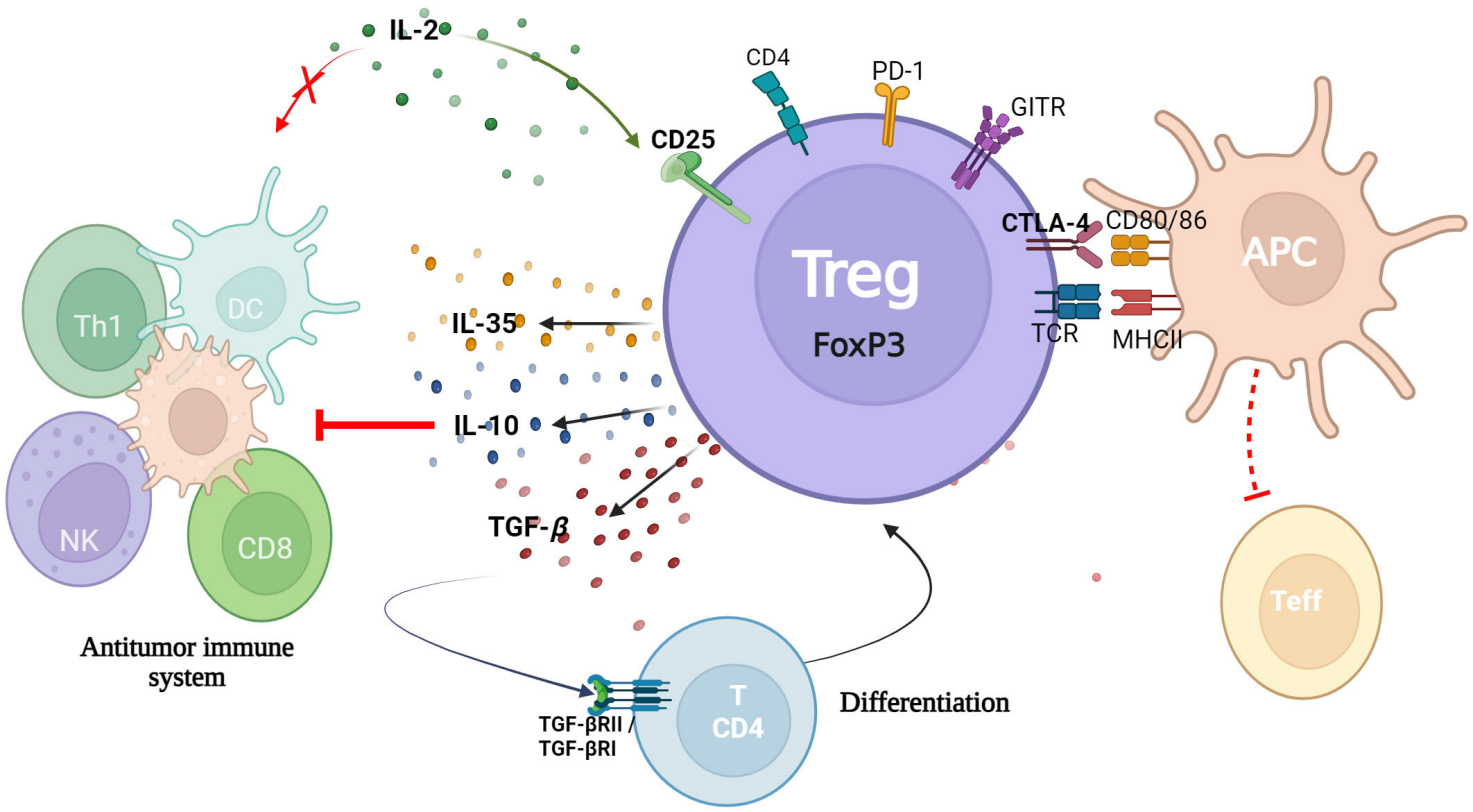
Mechanisms of Treg immunosuppression. Treg, characterized by the expression of the transcription factor FoxP3, exert multiple mechanisms of immune suppression. These include the secretion of immunosuppressive cytokines (IL-10, TGF-β, IL-35), the consumption of IL-2, inhibition of dendritic cell (DC) maturation, suppression of effector T cells (Teff), NK cells, CD8+ cells, and Th1 cells, as well as competition for co-stimulatory molecules (CD80/86) on antigen-presenting cells (APCs). They also express suppressive molecules such as CTLA-4 and LAG-3 and can induce apoptosis in effector immune cells, thereby promoting tumor immune evasion.

Regulatory T cells (Tregs) are a specialized subpopulation of CD4+ T cells with immunosuppressive activity, essential for immune system homeostasis, and the maintenance of immune tolerance. This cellular subtype was first identified in 1995 by Sakaguchi and colleagues (1). Tregs are characterized by the high expression of CD25, the IL-2 receptor α chain, which is crucial for their development, survival, and function (1, 2). Additionally, they express the transcription factor FoxP3, which is considered the master regulator of their identity and immunosuppressive activity (3, 4). Their regulatory capacity depends on several surface molecules with co-stimulatory and co-inhibitory functions. Among these, CTLA-4 (Cytotoxic T-Lymphocyte Antigen 4) stands out as it is constitutively expressed on Treg cells and exhibits high affinity for the costimulatory ligands CD80 and CD86 on antigen-presenting cells (APCs). By competing with CD28 on effector T cells, CTLA-4 interferes with their activation; through trans-endocytosis, it actively removes CD80/CD86 from the surface of APCs, further limiting their capacity to stimulate effector T cells (5). Similarly, PD-1 (Programmed Cell Death Protein 1), through its interaction with PD-L1 (Programmed Death-Ligand 1), initiates signaling that contributes to the maintenance of Foxp3 expression and prevents Treg dysfunction, as PD-1 deficiency can compromise both their stability and suppressive function (6, 7). In contrast, activation of GITR (Glucocorticoid-Induced TNFR-Related Protein) has been associated with reduced Foxp3 expression, impaired regulatory function, and decreased production of immunosuppressive cytokines such as IL-10 and TGF-β (8, 9). TIGIT (T cell immunoreceptor with Ig and ITIM domains), expressed on activated Tregs, supports Foxp3 stability and IL-10 secretion, thereby enhancing their suppressive profile (10, 11). Finally, ICOS (Inducible T-cell costimulator) is essential for sustaining Foxp3 expression via the PI3K-Akt-mTOR pathway; although TGF-β can induce Foxp3, ICOS is required to maintain its stable expression over time (12, 13). Collectively, these molecules modulate effector T cell activation and contribute to immune tolerance in various physiological and pathological contexts (7, 14, 15), **Figure 1**.

In the context of tumor immunity, Tregs restrain antitumor activity by directly suppressing cytotoxic lymphocytes. Through CTLA-4 and PD-1 engagement, they inhibit dendritic cell–mediated costimulation and limit CD8^+^ T cell priming, while IL-10 and TGF-β reduce the production of cytolytic molecules such as perforin and granzyme B (16). In addition, Tregs suppress natural killer (NK) cell function by downregulating NKG2D and CD107a expression through cell-contact-dependent mechanisms and enhanced TGF-β signaling (17, 18). These combined mechanisms blunt cytotoxic function and favor immune evasion within the tumor microenvironment. Through these mechanisms, Tregs play a crucial role in preventing the activation of autoreactive T cells and suppressing excessive or aberrant immune responses thus contributing to the stability of the immune environment (19, 20). The functional impact of Tregs is highly context-dependent, while their suppressive activity is essential for preventing autoimmunity, in the oncological setting, these same mechanisms can hinder effective antitumor responses, emphasizing the duality of Treg biology and the challenge of targeting them therapeutically.

## 2 Importance of Tregs in cancer and tumor microenvironment

In diseases such as cancer, regulatory T cells play a complex role in immune regulation by suppressing effector T cell responses within the tumor microenvironment. This activity can facilitate immune evasion and contribute to cancer progression (21). Across both solid and hematological malignancies, their presence correlates with tumor aggressiveness, therapy resistance, and poor clinical outcome. The mechanisms underlying this immunosuppressive influence are conserved and can be grouped into four main categories: recruitment and accumulation, cytokine-mediated suppression, immune checkpoint engagement, and induction of therapeutic resistance.

In triple-negative breast cancer, a high infiltration of Tregs has been observed in tumor tissues, playing a key role in tumor microenvironment immunosuppression, thereby promoting cancer progression and treatment resistance, including immunotherapies (22). It has been shown that the increased immunotherapy resistance may be due to the accumulation a of highly suppressive subtype known as effector Tregs (eTregs), characterized by the high expression of CD25 (IL-2Ra), FOXP3, and 4-1BB (CD137) (23). Functionally, eTregs represent an activated and terminally differentiated subset with enhanced metabolic activity and potent suppressive function, sustaining immune evasion and resistance to PD-1 blockade within the tumor microenvironment (23, 24).

A key feature of Treg accumulation in tumors is their recruitment and retention, driven by chemokine gradients, adhesion molecules, and local survival cues. Tumor cells and tumor-associated macrophages upregulate CCL22 and CCL17, ligands for CCR4, which promote Treg chemotaxis toward the tumor microenvironment (16, 25, 26). Beyond migration, Tregs are maintained within the tumor through local expansion, phenotypic stabilization, and metabolic adaptation. Tumor-derived metabolites, such as lactate and adenosine, sustain Treg suppressive function and promote FoxP3 stability, representing a key metabolic axis that supports their persistence in hypoxic conditions (27). Collectively, tumor-infiltrating Tregs display distinct chemokine receptor expression and metabolic plasticity compared to peripheral Tregs, enabling their long-term survival and immunosuppressive potency within the tumor microenvironment.

### 2.1 Recruitment and accumulation within the tumor microenvironment

Several cancers actively attract or expand Tregs through chemokine-mediated mechanisms. In epithelial ovarian cancer, for example, the chemokine CCL22 recruits CD4^+^CD25^+^FOXP3^+^ cells, leading to suppression of antitumor immunity and favoring tumor progression (28–30). Similarly, the loss of macroH2A1 in hepatocellular carcinoma cells increases CD4^+^CD25^+^FOXP3^+^ Tregs, promoting chemoresistance and immune evasion (31). In non-small cell lung cancer (NSCLC), the IDO1–kynurenine pathway fosters naïve T cell conversion into Tregs (32), while tumor-derived IL-37 polarizes macrophages toward a suppressive phenotype, correlating with elevated Treg presence and reduced NK activity (33). In head and neck squamous cell carcinoma (HNSCC), tumor endothelial cells release extracellular vesicles enriched in TGF-β1, inducing differentiation of naïve CD4^+^ T cells into Tregs and reinforcing immune suppression (34). Likewise, acute lymphoblastic leukemia (ALL) and acute myeloid leukemia (AML) show a proportional increase in circulating Tregs relative to tumor burden, emphasizing their systemic immunosuppressive role (35, 36).

### 2.2 Suppression via immunoregulatory cytokines

Tregs exert potent inhibition of effector immune cells through anti-inflammatory cytokines such as IL-10, TGF-β, and IL-35. In gastric cancer, Treg expansion correlates with regulatory B cells producing IL-10 and TGF-β, promoting immune tolerance and advanced disease (37). Single-cell analyses confirmed Treg enrichment in gastric tumors relative to healthy tissue (38). In B-cell lymphoma, elevated IL-10 and TGF-β from FOXP3^+^ Tregs enhance tumor progression and immune evasion (39). Furthermore, in AML, IL-35–producing Tregs activate STAT1/STAT3 signaling, promoting blast proliferation and resistance to apoptosis (36). Collectively, these cytokine-driven mechanisms maintain a suppressive milieu that favors tumor persistence and progression.

### 2.3 Immune checkpoint–mediated inhibition

Tregs express a broad range of checkpoint molecules that dampen cytotoxic and helper T cell function. In triple-negative breast cancer (TNBC), high infiltration of CD25^hi FOXP3^+^ 4-1BB^+^ effector Tregs (eTregs) is linked to immune evasion and resistance to PD-1 blockade therapies (23, 24), representing a terminally differentiated and metabolically active subset with strong suppressive capacity that sustains an immunosuppressive tumor milieu and limits antitumor T cell activity (24). Similarly, in HNSCC, CTLA-4^+^ Tregs expand following cetuximab treatment, suppressing NK cytotoxicity and correlating with poorer prognosis (40, 41). Esophageal carcinoma and NSCLC also exhibit increased PD-1, CTLA-4, and TNFR2 expression on Tregs, reinforcing their suppressive function (42–45). The expression of these molecules stabilizes FOXP3 and augments the suppressive capacity of Tregs, contributing to therapy resistance and immune escape across multiple cancers.

### 2.4 Promotion of therapeutic resistance

Tregs not only mediate baseline immunosuppression but also actively induce resistance to therapy. In TNBC, their enrichment reduces the efficacy of checkpoint inhibitors (22–24). In NSCLC, the kynurenine pathway and TNFR2 activation are associated with cisplatin resistance and metastasis (45). Similarly, in hepatocellular carcinoma, increased Tregs following macroH2A1 loss correlate with chemoresistance (31). These patterns highlight the role of Tregs as dynamic regulators of therapy response. Although glioblastoma shows inconsistent associations between Treg levels and survival (46), brain metastases demonstrate that increased Treg infiltration accompanies T cell exhaustion, supporting an immunosuppressive microenvironment (47). In bladder cancer, specific Treg subsets suppress MHC class II–dependent cytotoxic CD4^+^ T cells, further contributing to immune escape (32, 45, 48).

**Figure 2:**
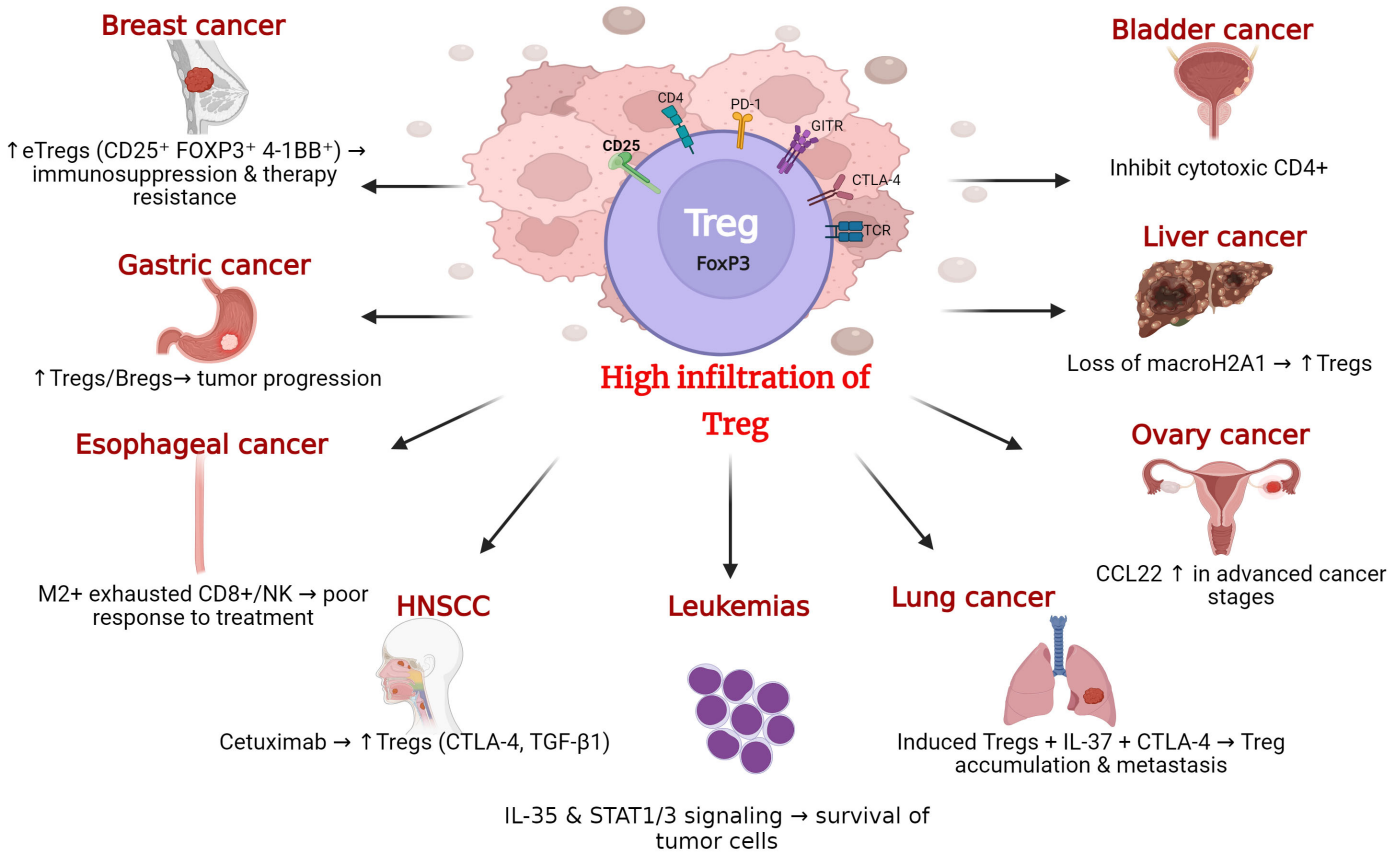
Treg lymphocytes in the tumor microenvironment across different cancer types. The figure shows a highly infiltrated Treg cell (FoxP3^+^) within the tumor microenvironment characterized by the expression of multiple immunoregulatory markers such as CD25, CTLA-4, PD-1, TCR, and GITR. This accumulation of Treg has been identified in numerous types of cancer, including breast, gastric, esophageal, bladder, liver, ovarian, and lung cancers, as well as in head and neck squamous cell carcinomas (HNSCC) and hematological malignancies such as leukemias. In all these cases, the high presence of Treg promotes an immunosuppressive environment that facilitates tumor immune evasion, cancer progression, and, in many cases, resistance to therapies, including immunotherapy.

In summary, studies across multiple tumor types—including lung (32, 44, 45), breast (23, 24) ovarian (28–30), gastric (37, 38), hepatocellular (31), and hematological malignancies (35, 36)—consistently demonstrate that increased Treg infiltration correlates with disease progression, treatment resistance, and poor prognosis, **Figure 2**, **Table 1**. However, it is evident that the extent to which Tregs contribute to tumor promotion varies across contexts, reflecting differences in their activation state, metabolic adaptation, and interaction with other immune cells. These findings underscore the conserved nature of Treg-driven immunosuppression and highlight their value as a therapeutic target to enhance the efficacy of current cancer immunotherapy.

**Table 1:** Treg-mediated immunosuppressive mechanisms across different cancer types.

Cancer type	Treg markers/subtype	Main mechanism	Clinical impact	Representative studies
Triple-negative breast cancer (TNBC)	CD25^hi FOXP3^+^ 4-1BB^+^ effector Tregs (eTregs)	Checkpoint-mediated suppression; PD-1 blockade resistance	Immunotherapy resistance	Miyara et al., 2009 (24); Fattori et al., 2023 (23)
Ovarian cancer	CD4^+^ CD25^+^ FOXP3^+^ Tregs	CCL22-mediated recruitment by tumor cells and TAMs	Poor treatment response; disease progression	Knutson et al., 2015 (28); Wertel et al., 2015 (29); Brtnicky et al., 2015 (30)
Gastric cancer	FOXP3^+^ Tregs/Regulatory B cells (IL-10, TGF-β)	Cytokine-mediated suppression	Poor prognosis; advanced stage	Wang et al., 2015 (37); Sathe et al., 2020 (38)
Esophageal carcinoma	FOXP3^+^ Tregs; PDCD1^+^; CTLA-4^+^	Checkpoint-mediated suppression; exhausted CD8^+^/NK cells	Poor response to therapy	Vacchelli et al., 2015 (42); Muto et al., 2015 (44)
Head and neck squamous cell carcinoma (HNSCC)	CTLA-4^+^ Tregs induced by cetuximab (TGF-β1 dependent)	Checkpoint-mediated suppression; EV-mediated induction	NK cell suppression; worse prognosis	Jie et al., 2015 (41); Lopatina et al., 2020 (34); Cillo et al., 2020 (40)
Non-small cell lung cancer (NSCLC)	FOXP3^+^ HELIOS^-^ TNFR2^+^ Tregs	IDO1–kynurenine axis; IL-37/IL-37R–MARCO pathway	Metastasis; cisplatin resistance	Yan et al., 2015 (45); Nguyen et al., 2020 (32); La Fleur et al., 2021 (33)
Glioblastoma	Variable Treg frequency	No clear correlation with outcome	Unclear association	Thomas et al., 2015 (46)
Brain metastases	Tregs with T-cell exhaustion signature	Immunosuppression/T cell exhaustion	Reduced effector function	Friebel et al., 2020 (47)
Bladder cancer	MHC-II suppressive Tregs	Inhibition of cytotoxic CD4^+^ T cells	Immune evasion; tumor persistence	Oh et al., 2020 (48)
Hepatocellular carcinoma (HCC)	FOXP3^+^ Tregs induced by macroH2A1 loss	Chemoresistance-associated recruitment and immune evasion	Tumor persistence/chemoresistance	Re et al., 2020
B-cell acute lymphoblastic leukemia (B-ALL)	High circulating Tregs	Systemic recruitment correlating with tumor burden	Poor prognosis (≥10 y old patients)	Idris et al., 2016 (35)
Acute myeloid leukemia (AML)	IL-35-producing FOXP3^+^ Tregs	Cytokine-driven STAT1/STAT3 activation	Apoptosis resistance; blast proliferation	Tao et al., 2015 (36)
B-cell lymphoma	FOXP3^+^ Tregs with IL-10/TGF-β expression	Cytokine-mediated suppression	Tumor progression and immune evasion	Wu et al., 2015 (39)

## 3 Targeted immunotherapies against Tregs

**Figure 3:**
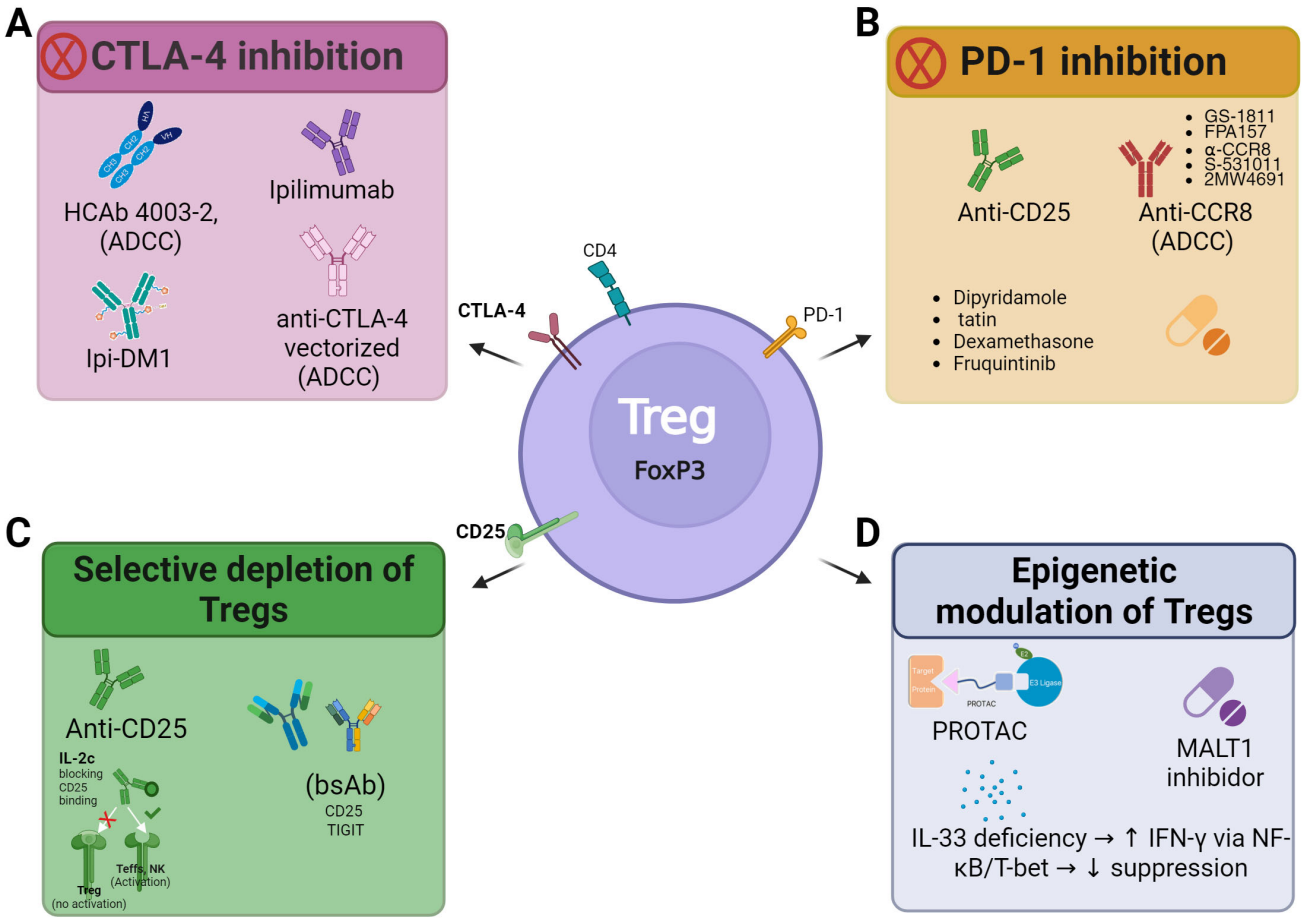
Immunotherapeutic strategies targeting treg to enhance antitumor response. Four main approaches are illustrated: **(A)** CTLA-4 inhibition, including antibodies such as ipilimumab and variants with antibody-dependent cellular cytotoxicity (ADCC) capability; **(B)** PD-1/PD-L1 blockade, using anti-CD25 antibodies, anti-CCR8 antibodies, and pharmacological compounds; **(C)** Selective depletion of Tregs, through anti-CD25 antibodies or bispecific antibodies (bsAb) that prevent Treg activation; and **(D)** Epigenetic modulation of Treg, using MALT1 inhibitors and protein degraders (PROTAC), including the impact of IL-33 deficiency, which promotes IFN-γ expression via NF-κB/T-bet signaling, thereby reducing their suppressive capacity.

Treg has become a possible target for tumor treatment and efforts to either reduce or deplete them from tumors are under profuse investigation. As shown in **Figure 3**, four main strategies have been designed, all of them considering either specific Treg surface receptors or determinants or using specific ubiquitin-promoting chimeric molecules that could then promote the proteosome-mediated degradation of important intracellular factors needed for Treg function. The clearest surface factors are CTLA-4, PD1, and CD-25 that could be used to either disrupt the availability of the surface determinant or can impair the Treg activation whereas proteasome-mediate degradation includes processes such as nuclear translocation, protein secretion and localization.

### 3.1 CTLA-4 inhibition

Strategies targeting CTLA-4 have proven effective in reducing Treg-mediated immunosuppression and enhancing antitumor responses. In colorectal cancer models, the combination of CTLA-4 inhibition with Treg depletion via Ipilimumab has improved therapeutic efficacy by reducing Treg presence within the tumor and promoting the activation of effector T cells (49).To optimize these strategies, innovative approaches such as antibody-drug conjugates have been developed. One such example is Ipilimumab-emtansine (Ipi-DM1), which significantly reduces Foxp3+CD4+ Tregs, decreasing CTLA-4 and Ki67 expression, thus favoring effector T cell expansion and a more active immune response (50), **Figure 3A**. Similarly, the use of FLT3 ligand (FLT3L) to increase the number of classical dendritic cells (cDCs), combined with Treg depletion through anti-CTLA-4, has demonstrated improved survival rates in melanoma models (51).

Moreover, more selective strategies have emerged, such as the heavy-chain antibody HCAb 4003-2, which specifically eliminates Tregs in colorectal carcinoma tumors without affecting peripheral tissues. This approach utilizes antibody-dependent cellular cytotoxicity (ADCC) mediated by NK cells and macrophages, thereby reducing immunosuppression without systemic toxicity (52). Additionally, vectorized anti-CTLA-4 antibodies have shown promising results in glioblastoma and colorectal carcinoma models, achieving more efficient Treg depletion through Fc optimization, enhancing ADCC, and promoting antigen cross-presentation, thus strengthening the antitumor immune response (53), **Figure 3A**. These advances highlight the potential of CTLA-4 blockade as a key strategy to overcome tumor-induced immunosuppression and enhance immunotherapy efficacy across several cancer types.

### 3.2 PD-L1 inhibition

Strategies based on PD-1/PD-L1 inhibition have also proven effective in enhancing immune responses against cancer by reducing Treg-mediated immunosuppression. In triple-negative breast cancer, the combination of anti-CD25 antibodies with PD-1 inhibitors reversed PD-1 blockade resistance by restoring a more active immune microenvironment (23). Similarly, the DBDx triple combination (Dipyridamole, Bestatin, Dexamethasone) potentiated anti-PD-1 efficacy by decreasing Treg infiltration and activity (54) **Figure 3B**, while the simultaneous inhibition of PD-1 and VEGFR-2 promoted an increase in cytotoxic T cells in murine models (55).

In colorectal cancer, the combination of Fruquintinib or AB680 with anti-PD-1 therapy reduced the presence of Tregs and promoted the infiltration of CD8^+^ and IFN-γ–producing CD4^+^ T cells. Notably, AB680 also favored the polarization of macrophages toward the M1 phenotype, thereby enhancing antitumor immune responses (56, 57). Additionally, FcγR stimulation with anti-hOX40 hIgG1 enhanced effector T cell activation and, when combined with PD-1 inhibitors, improved antitumor responses in preclinical models (58). In melanoma patients receiving immune checkpoint inhibitors such as anti-PD-1, the development of immune-related adverse events was associated with a transcriptomic reprogramming of Tregs, activating pro-inflammatory signaling pathways and metabolic alterations along with apoptotic signals, including IFNγ, IL-6, IL-1β, IL-33, TNFα, NF-κB complexes and transcription factors such as STAT1, STAT4, STAT5, IRF1, IRF3, IRF5, and IRF7. This indicates a reprogramming that promotes an exacerbated immune response rather than immunosuppression (59).Beyond their direct effects on Tregs, anti-PD-1 therapy can also influence immunosuppression mediated by tumor-associated macrophages, which induces Treg through PD-1. In murine models, its inhibition decreased Treg populations suggesting a promising strategy to restore immune surveillance (60).

Resistance to immune checkpoint inhibitors (ICIs) remains a challenge in oncology. A recent study in colorectal cancer (CT26) and lung cancer (TC1) murine models evaluated the combination of second-generation ICIs with Treg depletion and a tumor antigen-specific vaccine. In TC1, the combination of anti-TIGIT and anti-PD-L1 together with the vaccine induced a strong antitumor response, while Treg depletion using CD4+ T cell-targeting antibodies further enhanced this effect (61). In glioblastoma, glutamate transport has been implicated in resistance to anti-VEGF therapy by enhancing Treg function. However, CD25 blockade combined with anti-VEGF treatment restored CD8+ T cell IFNγ production and improved antitumor responses (62).

Another innovative strategy involves anti-CCR8 antibodies targeting a Treg-specific marker in the tumor microenvironment. GS-1811, FPA157, and α-CCR8, engineered for enhanced FcγR interaction, selectively depleted Tregs without affecting peripheral tissues such as the spleen, thymus, or skin, while increasing cytotoxic T cell activity (63). Furthermore, combining anti-CCR8 with PD-1 inhibitors yielded synergistic effects, improving antitumor responses (64, 65) **Figure 3B**. The humanized monoclonal antibody S-531011, specific for CCR8, has been evaluated in murine colorectal carcinoma (CT26.WT) and breast carcinoma (EMT6) models, reinforcing tumor immunity through selective intratumoral Treg depletion. Its combination with PD-1 inhibitors could further enhance antitumor responses (66). Another tetravalent symmetric antibody, 2MW4691, targets CCR8, a tumor-resident Treg-specific marker, with low CTLA-4 affinity, eliminating intratumoral Tregs via ADCC without compromising CD8+ T cell activation. This resulted in significant tumor growth reduction, combining Treg depletion with effective CD8+ T cell activation, presenting an innovative approach to improve immunotherapy efficacy in solid tumors (67).

These findings underscore the importance of combining immune checkpoint inhibition with Treg-targeting strategies to enhance immunotherapy efficacy. The **Table 2** summarizes the experimental models, cancer types, and immunotherapeutic strategies targeting modulation of Treg cell activity in various tumor contexts. Murine and human studies are included to illustrate the translational potential of these approaches.

**Table 2:** preclinical and clinical strategies targeting regulatory T cells in cancer immunotherapy.

Type of cancer	Experimental model	Therapy	Reference
Glioblastoma	Xenograft of U87MG or glioblastoma stem-like cells in athymic nude mice or NOD-scid IL2Rγnull mice.	CD25 blockade, anti-VEGF treatment (bevacizumab).	Long, Y. et al. (2020) (62)
Colon	Immunocompetent model CT26 in BALB/c mice	AB680 (a CD73 inhibitor) and anti-PD-1 antibodies.	Kim, M. et al. (2021) (57).
	Xenografts of HCT116 or SW620, typically MSS in NSG or nude mice. Immunocompetent model C57BL/6 mice with MC38 cells	Combined use of fruquintinib and anti-PD-1 antibodies	Wang, Y. et al. (2020) (56).
	Immunocompetent model C57BL/6 mice with MC38 cells	Vectorized anti-CTLA-4	Semmrich, M. et al. (2022) (53)
	Immunocompetent model Colon carcinoma MC38	9D9 (anti-CTLA-4 antagonist), b1s1e2-Fc (non-antagonistic variant), and LALA-PG Fc-silenced versions	Lax, B. M. et al. (2023) (49).
	Immunocompetent model Human CTLA-4 knock-in transgenic C57BL/6 mice implanted with MC38 or CT26 cells	CTLA-4-targeting heavy-chain-only antibodies (HCAb 4003-1 and HCAb 4003-2)	Gan, X. et al. (2022) (52)
	Syngeneic mouse tumor models: EG.7-OVA in hOX40 knock-in mice and CT26 cells	Anti-OX40 antibodies, PD-1 inhibitors	Willoughby, J. E. et al. (2024) (58).
	Murine BALB/c (CT26) and C57BL/6 (TC1) tumor models, including FoxP3-GFP^DTR^ transgenic mice	Anti-TIM-3, LAG-3, TIGIT, KLRG1 (CT26/TC1); E7 vaccine (TC1); Treg depletion (DT)	Becker, W. et al. (2024) (61).
Melanoma	Immunocompetent model B16F10 melanoma in Foxp3^DTR^ transgenic mice	Combination of diphtheria toxin and anti-CTLA-4 antibody	Lax, B. M. et al. (2023) (49).
	Immunocompetent model C57BL/6 mice with B16 melanoma	FLT3-L, anti-CTLA-4, or Treg depletion	Régnier, P. et al. (2023) (51)
	Immunocompetent model B16 melanoma murine models	Combination of anti-PD-1 antibody with DBDx triple therapy (Dipyridamole, Bestatin, Dexamethasone)	Shan, C. et al. (2023) (54).
	Patients with advanced metastatic melanoma and several cancers and autoimmune diseases	Anti-PD-1	Grigoriou, M. et al. (2021) (59).
Lymphoma	Autoimmune deficiency model: Human CTLA-4-expressing knock-in mice (hCTLA-4 KI)	Ipilimumab conjugated to the cytotoxic agent DM1 (Ipi-DM1)”	Muthana, M. M. et al. (2023 (50)
Breast	Spontaneous murine breast cancer models	Tconv culture with TAM supernatants, PD-1 inhibition	Kos, K. et al. (2022) (60).
	Patients with TNBC and murine TNBC synergistic model	PD-1 blockade, optimized anti-CD25 antibodies	Fattori, S. et al. (2023) (23).
	Immunocompetent murine models: BALB/c mice with 4T1 breast cancer	Monotera, pia anti-PD-1, **DBDx**: (*Dipyridamole,Bestatin,Dexamethasone*)	Shan, C. et al. (2023) (54).
	Spontaneous murine breast cancer models	Tconv culture with TAM supernatants, PD-1 inhibition CRISPR-PD-1 knock-out, anti-CSF1R antibodies	Kos, K. et al. (2022) (60).
Lung	Immunocompetent models using C57BL/6 mice bearing Lewis lung carcinoma (LLC).	Monotera, pia anti-PD-1, **DBDx**: (*Dipyridamole,Bestatin,Dexamethasone*)	Shan, C. et al. (2023) (54).
Hepatocellular carcinoma	Orthotopic and induced murine HCC models: HCA-1 implanted in C3H mice, RIL-175 (p53/Hras-driven) in C57BL/6 mice, plus a genetically engineered model carrying Mst1/2 mutations.	Simultaneous inhibition of PD-1 and VEGFR-2	Shigeta, K. et al. (2020) (55).

### 3.3 Selective Treg depletion

Selective elimination of Tregs within the tumor microenvironment is a key strategy to enhance immune responses against cancer. A promising approach involves non-IL-2-blocking anti-CD25 antibodies, which allow to maintain the activation of effector T cells (Teffs). The combination of these antibodies with CTLA-4 blockade has shown synergistic effects, improving therapeutic efficacy without compromising Teffs function (68), **Figure 3C**.

Optimized antibodies such as RG6292 have been designed to selectively bind CD25 on Tregs, allowing IL-2 to continue stimulating Teffs. Preclinical studies in non-human primates and humanized murine models demonstrated efficient Treg depletion without evident immunological toxicity, supporting its potential in combination therapies (69). Another optimized therapeutic antibody, anti-CD25-F(ab’)2 NIR-PIT, effectively depleted intratumoral Tregs in murine colorectal and breast cancer models without affecting peripheral Tregs. This resulted in rapid activation of antitumor CD8+ and NK cells, reducing the immunosuppressive capacity of the tumor microenvironment (70). Similarly, in primary breast cancer and lung metastasis murine models, anti-CD25-mediated Treg depletion was more effective in primary tumors than in metastatic control (71). In melanoma and sarcoma models, Treg depletion or CD4+ T cell transfer in lymphodepleted animals increased GzmB expression in tumor-infiltrating CD4+ T cells (72).

In chronic lymphocytic leukemia, a similar pattern has been observed where Treg depletion and IL-10 neutralization partially restored neutrophil function, suggesting that targeting Treg-associated signaling pathways could improve immune responses in these patients (73). Despite their potential, Treg depletion strategies do not always yield expected outcomes. In pancreatic cancer models, Treg depletion failed to alleviate immunosuppression and instead altered the tumor microenvironment, accelerating disease progression. This underscores the complexity of Tregs in cancer and the necessity for refined therapeutic approaches (74).

Additionally, CD122-selective IL-2 complexes (IL-2c), combined with PD-L1 blockades have shown efficacy in reducing Treg-mediated immunosuppression and enhancing IL-2 signaling in effector T cells, improving antitumor responses (75). The Bempegaldesleukin IL-2 agonist (NKTR-214) preferentially activates the CD122 (IL-2Rβ) receptor and, when combined with checkpoint blockade therapy, reduces intratumoral Tregs while expanding effector CD8+ T cells, potentiating immune responses (76). A bispecific antibody (bsAb) targeting CD25 and TIGIT, named NSWm7210 in murine models and NSWh7216 in human models, has also been developed. These antibodies have demonstrated selective depletion of intratumoral Tregs through antibody-dependent cytotoxicity, thus enhancing effector T cell activation and significantly inhibiting tumor growth in preclinical models. Furthermore, this strategy minimizes the depletion of peripheral Tregs, reducing the risk of autoimmunity and preserving systemic immune tolerance (77), **Figure 3C**. These Treg depletion strategies using either selective antibodies, IL-2 agonists, or immune checkpoint inhibitors stand out as key approaches to improving the efficacy of immunotherapy in cancer treatment, **Figure 3C**.

### 3.4 Epigenetic modulation of Tregs

The epigenetic regulation of Tregs has emerged as a key strategy for modulating immune responses in cancer. FoxP3, the transcription factor essential for Treg function, is regulated by epigenetic modulators such as ubiquitin-specific peptidase 22 (Usp22) and ring finger protein 20 (Rnf20). Studies in murine models with Usp22 deletion showed a decreased FoxP3 stability, reducing the immunosuppressive activity of Tregs and promoting a stronger antitumor response, suggesting its potential as a therapeutic target in cancers such as melanoma and colorectal cancer (78).

Among the most innovative strategies is the targeted degradation of FoxP3 using PROTACs (proteolysis targeting chimeras), which allows for precise modulation of the tumor microenvironment. This approach promotes effector T cell infiltration and enhances the response to immunotherapies without compromising systemic immune homeostasis (79). The use of PROTACs has emerged as a promising strategy to selectively modulate key Treg proteins such as FoxP3. Employing a 15-mer peptide that binds FoxP3, a click-chemistry–based linker, and the von Hippel–Lindau (VHL) E3 ligase, Yang and co-workers effectively inhibited FoxP3 nuclear translocation and promoted its subsequent degradation at concentrations as low as 1 µM. This targeted degradation of FoxP3 exemplifies how PROTAC-based approaches could reprogram Treg activity, potentially enhancing antitumor immune responses without inducing systemic autoimmunity (80) **Figure 3D**.

Another promising strategy is the inhibition of MALT1, which reduces Treg proliferation and suppresses IL-2 secretion, suggesting its potential as an immunotherapeutic approach in solid tumors (81). Additionally, interleukin 33 (IL-33) has been shown to regulate Tregs in the context of cancer. IL-33 deficiency in these cells impairs their suppressive capacity by inducing epigenetic reprogramming that increases IFN-γ expression in an NF-κB and T-bet-dependent manner, which alters the tumor immune response (82) **Figure 3D**.

## 4 Immunotherapies that indirectly modulate Tregs

It has been demonstrated that non-ablative oligofractionated irradiation induces a significant increase in Tregs within the tumor microenvironment in malignant mesothelioma. However, combining this irradiation with selective Treg depletion enhances the antitumor response both locally and systemically (83). A similar approach, using a single ablative dose followed by low-dose fractionation, resulted in the reprogramming of the tumor microenvironment, reducing immunosuppression by decreasing the presence of Tregs in irradiated tumors and secondary lymphoid organs. This Treg reduction was accompanied by increased infiltration of IFN-γ-producing CD8+ and CD4+ T cells, suggesting a more effective immune response against the tumor (84). Additionally, combining Foxp3+ Treg depletion with non-ablative oligofractionated irradiation improved local tumor control and generated systemic immune responses in murine models of malignant mesothelioma (83).

The role of Tregs in tumor resistance to combine radiotherapy and immune checkpoint blockade therapies has been extensively analyzed. It has been observed that eliminating or functionally inhibiting Tregs can enhance therapeutic responses in this context, increasing the efficacy of radiotherapy combined with dual immune checkpoint blockade in melanoma models (85). Moreover, combined therapies involving radiotherapy, Treg depletion with anti-CD25, and CD137 agonism have proven to be key in generating an effective T cell response in highly radioresistant tumors, inducing a phenotypic shift in Tregs, which acquire effector-like characteristics such as the production of IFN-γ and TNF-α (86).

The modulation of Treg in the tumor microenvironment has emerged as one of the promising strategies in cancer immunotherapy. Metabolic therapies targeting Treg aim to alter their metabolism to reduce their suppressive capacity and enhance the antitumor response. The strategies highlight the potential to modify Treg metabolism to reprogram their function reducing their suppressive capacity and enhancing the antitumor response in several types of cancer. In colorectal cancer, the suppression of the MondoA-TXNIP axis alters Treg metabolism, compromises their suppressive ability and enhances the antitumor immune response (87). In breast cancer, the inhibition of the Hedgehog (Hh) signaling pathway regulates Treg plasticity, promoting their immunosuppressive differentiation. Blocking this pathway metabolically reprograms Tregs reducing their suppressive capacity and favoring their conversion into inflammatory Th17 cells. This change improves the tumor microenvironment by increasing the recruitment of cytotoxic CD8+ T cells and opening new therapeutic possibilities (88). In ovarian cancer, the use of denileukin diftitox (DD) in combination with interferon-alpha (IFN-α) leads to a reduction in Treg and the reprogramming of their function towards a less suppressive phenotype both in murine models and in patients (89).

## 5 Cellular plasticity as an immunotherapeutic strategy

**Figure 4:**
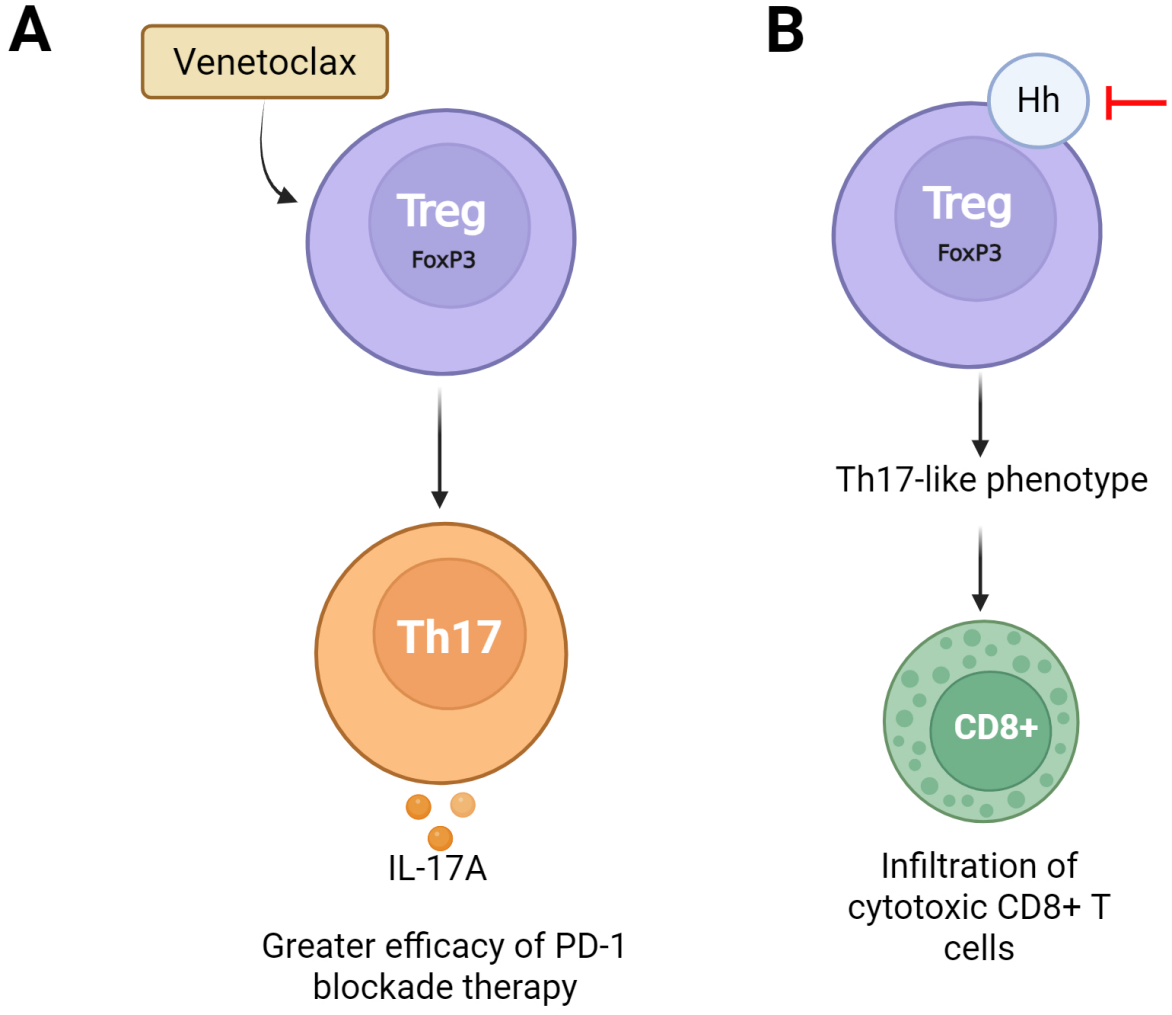
Modulation of Treg phenotype in the tumor context through pharmacological strategies. **(A)** Treatment with Venetoclax, a BCL-2 inhibitor, promotes the phenotypic shift of Treg cells (FoxP3^+^) toward a Th17 profile, associated with a proinflammatory environment. **(B)** Inhibition of the Hedgehog **(Hh)** signaling pathway in Treg cells enhances the effector function of CD8^+^ T cells, contributing to a more effective antitumor response. These strategies highlight the therapeutic potential of reprogramming Treg cells to overcome immunosuppression in the tumor microenvironment.

Treg plasticity is an emerging phenomenon in oncological research as these cells can adapt and differentiate into other T cell subtypes in response to tumor microenvironment signals. This reprogramming capacity directly influences tumor progression and represents a novel therapeutic target (90). Understanding the mechanisms regulating Treg plasticity could enable the development of strategies to reprogram or inhibit their immunosuppressive function in the tumor microenvironment (88). The plasticity has been recognized as a key strategy for improving the efficacy of immunotherapy in cancer treatment. Inhibition of BCL-2 using Venetoclax, for example, induces Treg plasticity towards a Th17-like phenotype increasing IL-17A production in lymphoid organs and the tumor microenvironment. This change promotes the conversion of Treg into more inflammatory cells, enhancing the effectiveness of PD-1 blockade therapy and suggesting that Venetoclax could offer therapeutic benefits in several malignancies by modulating the immune response (90), **Figure 4**.

Additionally, the Hedgehog (Hh) signaling pathway regulates Treg plasticity in breast cancer promoting their differentiation and immunosuppressive activity. However, inhibiting Hh reprograms Treg by reducing their suppressive capacity and favoring their conversion into inflammatory Th17 cells. This shift enhances cytotoxic CD8+ T cell recruitment and opens new therapeutic possibilities for breast cancer treatment (88), **Figure 4**.

The study of various Treg molecules that can modify their phenotype, or functionality, such as Toll-like receptors (TLRs), should be further explored in cancer pathologies (83, 85, 91). More detailed investigations are needed to understand the underlying mechanisms regulating immune cell interactions during systemic TLR stimulation in cancer. These findings suggest that combining Treg-targeted therapies with strategies that modulate their plasticity, and metabolism could significantly improve the efficacy of cancer immunotherapies.

Another dimension of Treg plasticity involves the emergence of Th1-like or “fragile” Tregs, which retain FOXP3 expression but exhibit reduced suppressive capacity and begin to secrete IFN-γ under inflammatory or therapeutic conditions. This conversion is driven by activation of the IFN-γ–STAT1 axis, which partially destabilizes the Treg transcriptional program and shifts these cells toward a pro-inflammatory phenotype (92). Fragile Tregs have been observed within tumor-infiltrating lymphocytes and are associated with improved antitumor immunity, as they promote immune activation rather than suppression (93). Similarly, reprogramming induced by anti-GITR or checkpoint blockade therapies can generate Th1-like Tregs that enhance cytotoxic T cell function and reshape the tumor microenvironment toward immune stimulation (94). Conceptually, this subset reflects a transitional state where Tregs lose their full regulatory stability, providing a potential therapeutic opportunity to convert suppressive Tregs into pro-inflammatory effectors (95).

## 6 Optimization of strategies to eliminate Tregs without affecting immune homeostasis

Selective elimination of Tregs in the tumor microenvironment is essential to enhance the efficacy of immunotherapies without inducing systemic adverse effects. One promising strategy is the use of optimized monoclonal antibodies, such as BAY 3375968, targeting CCR8. The Fc region modification enhances its ability to induce antibody-dependent cellular cytotoxicity (ADCC), allowing selective Treg depletion and improving interaction with immune effector cells, such as macrophages and NK cells (96). In breast cancer models, such as 4T1 and MDA-MB-231, traditional Chinese medicine, specifically Aiduqing, has been shown to inhibit metastasis by suppressing Treg infiltration induced by tumor-associated macrophages (TAMs) through the inhibition of the CXCL1 chemokine (97). Similarly, the NKG2D-Fc fusion protein significantly reduces Tregs and myeloid-derived suppressor cells, favoring NK cell infiltration into the tumor (98).While these antibody-based and combinatorial approaches demonstrate strong preclinical efficacy, clinical translation remains limited by potential off-target effects, partial depletion, and the lack of selective biomarkers distinguishing suppressive from homeostatic Tregs.

Another key strategy is Treg depletion using anti-CD25 antibodies, such as RG6292, designed to eliminate them without affecting effector T cells. Phase I clinical studies have shown that RG6292 is well tolerated and reduces Tregs in both peripheral circulation and the tumor microenvironment, though its efficacy in monotherapy or in combination with atezolizumab has been insufficient to overcome tumor resistance mechanisms, suggesting the need for additional therapeutic combinations (99). In this context, the combination of GITR agonism with Fc-γ receptor-mediated Treg depletion has been shown to increase the effector T cell-to-Treg ratio within tumors, promoting tumor reduction (100).

In addition to antibody-based and combinatorial approaches, other innovative strategies aim to modulate Treg function and enhance effector activation through cytokine and metabolic pathways. IL-2 engineering represents one of the most promising examples: the pegylated IL-2 variant THOR-707, designed to reduce CD25 binding while preserving IL-2Rβ/γ signaling, preferentially expands CD8^+^ T cells and NK cells over Tregs (101). Its clinical analogue, SAR-444245, showed similar selectivity and safety in primates, boosting cytotoxic activity without eosinophilia or vascular leak (102). Together, these engineered IL-2 variants highlight the therapeutic potential of cytokine redesign to strengthen antitumor immunity while limiting regulatory T-cell expansion. Despite encouraging preclinical results, long-term data on safety, durability of responses, and optimal dosing regimens remain scarce. These challenges underscore the importance of integrating IL-2 variants with immune checkpoint inhibitors or metabolic modulators to achieve sustained tumor control.

Low-dose cyclophosphamide (mCTX) also modulates Tregs by selectively depleting circulating and intratumoral subsets, restoring effector function. In neuroblastoma and breast cancer, mCTX reduced Treg density and improved tumor control (103, 104). Combined with checkpoint blockade, mCTX enhanced CD8^+^ T-cell activation and tumor regression in hepatocellular and other solid tumors (105, 106). Collectively, these findings highlight the immunomodulatory potential of low-dose mCTX as an adjuvant strategy to enhance antitumor responses while minimizing systemic toxicity.

Finally, metabolic and signaling targeting of Tregs via PI3Kδ inhibition provides a complementary route to weaken intratumoral immune suppression while preserving effector function. In patients with solid tumors, intermittent PI3Kδ inhibition selectively restrained Tregs, increased the CD8^+^:Treg ratio, and sustained antitumor immunity with manageable immune-related adverse events, supporting a dosing paradigm that decouples efficacy from toxicity (107). Preclinical and translational studies further demonstrate that blocking PI3Kδ disrupts Treg maintenance and reduces tumor burden while sparing cytotoxic T-cell function (108). Next-generation, highly selective inhibitors—such as BGB-10188 and IOA-244—have shown reduced Treg proliferation and potent antitumor activity *in vivo* and in early clinical trials, confirming PI3Kδ as a tractable Treg-centric target (109, 110).

Selective IL-2 deprivation in Tregs using anti-IL-2Rα (CD25) monoclonal antibodies combined with CD40 receptor activation has demonstrated synergistic effects, maintaining durable immune responses in lymphoma models and highlighting the potential of these therapies in tumor microenvironment modulation (111). Additionally, BAT6026, a monoclonal antibody targeting OX40, has been shown to reduce Treg populations in the tumor microenvironment and activate effector T cells, particularly CD8+, suggesting its combination with immune checkpoint inhibitors, such as anti-PD-1, to generate durable responses in murine cancer models (112).

A limiting factor for the efficacy of anti-Treg antibodies is the expression of FcγRIIB in the tumor microenvironment, which impairs efficient depletion of these cells. FcγRIIB inhibition has been shown to restore Treg depletion capacity and enhance the antitumor activity of antibodies (113). In a clinical study involving patients with several solid tumors, including esophageal, lung, malignant melanoma, gastric, ovarian, and mesothelioma cancers, the KW-0761 antibody effectively modulated Tregs. By blocking CCR4 signaling, KW-0761 reduced Treg infiltration in tumors, suggesting its potential as a therapeutic tool in oncology (114). Furthermore, in hepatocellular carcinoma, CCR4+ Tregs were observed and therapies targeting CCL22 (primary ligand of CCR4), using an antagonist or N-CCR4-Fc, blocked the accumulation of intratumoral CCR4+ Tregs, overcoming sorafenib resistance and sensitizing tumors to PD-1 (115).

Altogether, while Treg-targeted interventions continue to evolve rapidly, their long-term clinical success will depend on achieving selective modulation without disrupting systemic immune homeostasis—a balance that remains one of the main frontiers in cancer immunotherapy. To this end, several strategies have been developed to selectively eliminate Tregs within the tumor microenvironment while preserving global immune equilibrium. **Table 3** summarizes the main agents, mechanisms of action, and observed effects reported in preclinical and clinical models.

**Table 3:** Therapeutic strategies for selective depletion of Tregs in the tumor.

Strategy/agent	Mechanism of action	Type of study	Reference
BAY 3375968 (anti-CCR8 mAb)	Monoclonal antibody with optimized Fc region to induce ADCC and selectively deplete Tregs	Preclinical (Mechanistic and translational therapeutic)	Roider HG, et al., 2024 (96).
Aiduqing (traditional Chinese medicine)	Inhibits CXCL1 induced by TAMs, reducing Treg infiltration	Preclinical (Therapeutic/mechanistic)	Li J, et al., 2021 (97).
NKG2D-Fc (fusion protein)	Reduces Tregs and MDSCs; promotes NK cell infiltration into tumors	Preclinical (Mechanistic/immunotherapeutic)	Feng PH, et al., 2020 (98).
RG6292 (anti-CD25 mAb)	Depletes Tregs without ascffecting effector T cells	Clinical, 2 phase I trials (Assessment of safety, pharmacokinetics, pharmacodynamics, and antitumor activity)	Gambardella V, et al., 2025 (99).
GITR agonist + Treg depletion via FcγR	Effector T cell activation + Treg depletion mediated by Fcγ receptors	Preclinical (computational model)	Ji Y, et al., 2023 (100).
Anti-IL-2Rα + CD40 activation	Selective IL-2 deprivation in Tregs + immune activation through CD40	Preclinical (Mechanistic and preclinical therapeutic)	Stirm K, et al., 2023 (111).
BAT6026 (anti-OX40 mAb)	Reduces intratumoral Tregs and activates CD8+ T cells	Preclinical (Mechanistic and translational therapeutic)	Liang S, et al., 2023 (112).
FcγRIIB inhibition	Restores Treg depletion capacity by blocking inhibitory FcγRIIB receptor	Preclinical (Therapeutic/mechanistic)	Knorr DA, et al., 2024 (113).
KW-0761 (anti-CCR4 mAb)	Blocks CCR4 signaling to reduce Treg tumor infiltration	Clinical phase Ib trial in humans (Assessment of safety and clinical responses)	Saito T, et al., 2021 (114)
CCL22 antagonist or N-CCR4-Fc	Blocks accumulation of CCR4+ Tregs in tumors	Preclinical (Mechanistic and translational)	Gao Y, et al., 2022 (115).
THOR-707 (pegylated IL-2 variant)	Engineered IL-2 analog with reduced CD25 binding and preserved IL-2Rβ/γ signaling; selectively expands CD8^+^ T cells and NK cells over Tregs	Preclinical/Translational (Mechanistic and safety evaluation)	Ptacin E., et al., 2021 (101)
SAR-444245 (clinical-grade IL-2 analog)	Maintains IL-2Rβ/γ selectivity; enhances cytotoxic T and NK cell activation without eosinophilia or vascular leak	Preclinical (Nonhuman primate)	Ma L., et al., 2024 (102)
Low-dose Cyclophosphamide (mCTX)	Selectively depletes circulating and tumor-infiltrating Tregs; restores CD8^+^ and NK cell cytotoxicity	Preclinical and Clinical (Neuroblastoma, Breast, HCC models)	Webb M., et al., 2022 (104)
PI3Kδ Inhibitors (BGB-10188, IOA-244)	Inhibit Treg survival and proliferation while preserving effector T-cell function; increase CD8^+^:Treg ratio	Preclinical and Clinical (Translational immuno-oncology)	Eschweiler S., et al., 2022 (107)

## 7 Discussion

Tregs play a pivotal role in maintaining immune tolerance and homeostasis (1, 2). However, in the oncological setting, they significantly contribute to the establishment of an immunosuppressive tumor microenvironment, facilitating tumor progression and resistance to therapy, including immunotherapy (21, 22). This negative prognostic impact is further supported by a comprehensive meta-analysis encompassing 76 studies and over 15–000 patients, which demonstrated that elevated FOXP3^+^ Treg infiltration correlates with significantly reduced overall survival across multiple tumor types (116).

Beyond Treg abundance alone, the intra-tumoral CD8^+^/FOXP3^+^ (or CD8^+^/Treg) ratio has shown a stronger prognostic and predictive value across cancers. For instance, in colorectal cancer, a higher CD8^+^/FOXP3^+^ ratio was an independent predictor of improved overall survival when assessed by immunohistochemistry (117). More recently, in invasive breast cancer, patients with a high CD8^+^/eTreg ratio achieved significantly higher pathological complete response (pCR) rates following neoadjuvant chemotherapy compared with those with low ratios (118). Similarly, in triple-negative breast carcinoma, a high CD8^+^ to FOXP3^+^ ratio in the tumor stroma correlated with improved overall survival (119). Collectively, these findings underscore that the balance between cytotoxic and regulatory T cells—rather than Treg abundance alone—provides a more refined biomarker of antitumor immunity and therapeutic responsiveness. Selective depletion of intratumoral Tregs has emerged as a promising strategy to enhance antitumor immune responses without compromising peripheral tolerance. Anti-CD25 antibodies that preserve IL-2 signaling have shown synergistic effects when combined with CTLA-4 blockade (68); meanwhile, optimized formulations such as RG6292 and anti-CD25-F(ab’)_2_ NIR-PIT have demonstrated effective depletion of intratumoral Tregs in preclinical models without inducing systemic immune toxicity (69, 70). Moreover, antibodies targeting CCR8 such as GS-1811, S-531011, and 2MW4691 allow for highly specific depletion of tumor-resident Tregs via antibody-dependent cellular cytotoxicity (ADCC), promoting the expansion of CD8^+^ T cells and improving tumor control (63–67). Nevertheless, the role of Tregs is highly context-dependent, in certain cancers, such as pancreatic carcinoma, Treg depletion has paradoxically exacerbated disease progression, highlighting the need for more refined and tumor-specific therapeutic approaches (74).

Indirect modulation of Tregs, including non-ablative fractionated radiotherapy, has also proven effective in reprogramming the tumor microenvironment. This approach reduces Treg frequency while enhancing the infiltration of IFN-γ–producing CD8^+^ and CD4^+^ T cells, thereby potentiating both local and systemic antitumor immune responses (83, 85). Beyond radiotherapy, emerging evidence reveals that the microbiome, tumor-derived metabolites, and innate receptor signaling collectively shape Treg metabolism and function. Commensal bacteria such as Bacteroides fragilis and Clostridium species promote FOXP3^+^ Treg differentiation through short-chain fatty acids (SCFAs), particularly butyrate and propionate, which enhance histone acetylation at the FOXP3 locus and sustain suppressive stability (120, 121). Likewise, tumor-associated metabolites such as kynurenine, lactate, and adenosine maintain Treg function through activation of AhR and HIF-1α signaling (27, 122). Importantly, activation of Toll-like receptor 8 (TLR8) has been shown to reprogram Treg metabolism, inhibiting glucose uptake and glycolysis while downregulating the mTOR pathway, ultimately reducing their suppressive capacity (123–125). Together, these findings highlight a dynamic microbiota–metabolite–TLR axis that governs Treg metabolic adaptation and may represent a promising target for attenuating Treg-mediated immune suppression in cancer.

The phenotypic plasticity of Tregs represents another critical therapeutic target. Agents such as Venetoclax have been shown to induce a shift toward a Th17-like, proinflammatory phenotype, thus enhancing the efficacy of PD-1 blockade (90). Similarly, inhibition of the Hedgehog (Hh), Th17-like cell increasing CD8^+^ T cell recruitment and strengthening antitumor responses (88). The emergence of Th1-like, which retain FOXP3 expression but secrete IFN-γ under inflammatory or therapeutic stress, exemplifies this plasticity. This reprogramming, driven by IFN-γ–STAT1 signaling, destabilizes the Treg transcriptional program and shifts them toward a pro-inflammatory phenotype associated with improved antitumor immunity (93, 94). Reprogramming induced by anti-GITR or checkpoint blockade can likewise generate Th1-like Tregs that amplify cytotoxic responses and remodel the TME toward immune activation (95, 96). These “fragile” Tregs thus represent a transitional state in which suppressive cells acquire effector traits, offering opportunities to convert immune suppression into activation.

Integration of Treg-targeted strategies—including selective depletion, epigenetic modulation, metabolic reprogramming, and induction of cellular plasticity—with immune checkpoint blockade represents one of the most promising avenues for improving cancer immunotherapy. Such combined approaches aim to reshape the tumor microenvironment toward a more inflammatory and immunologically active state, restoring immune surveillance and responsiveness (49, 115). Nonetheless more selective approaches are needed to eliminate Tregs in tumors without affecting systemic immune homeostasis, combining Treg-targeted therapies with metabolic and epigenetic modulators could offer additional benefits enhancing therapeutic precision and durability. Future progress will depend identifying rubost biomarkers of Treg function and specificity in the tumor microenvironment. Biomarkers such as FcγRIIB, CCR4/CCL22, and especially the CD8^+^/Treg ratio may guide the stratification of patients most likely to benefit from Treg-targeted interventions. Parallel studies should also expand into hematologic malignancies, where Tregs influence disease progression and interact uniquely with leukemic cells, potentially unveiling new therapeutic opportunities.

## 8 Conclusion

The findings presented in this review highlight the fundamental role of Tregs in cancer progression and resistance to immunotherapy. Tregs in the tumor microenvironment contribute to immune evasion and represent a significant obstacle to the efficacy of immunotherapy. However, recent advances in Treg modulation, including selective depletion strategies, phenotypic reprogramming (also known as plasticity), and epigenetic regulation, offer new therapeutic opportunities. Among these cellular plasticity has emerged as an innovative concept in cancer immunotherapy, allowing Tregs to undergo functional reprogramming toward less suppressive or even proinflammatory phenotypes. Experimental evidence indicates that BCL-2 inhibition with Venetoclax and Hedgehog (Hh) pathway suppression in breast cancer can induce Treg conversion toward a Th17-like phenotype, reducing their immunosuppressive capacity, promoting effector cell infiltration, and amplifying antitumor responses. These findings highlight the potential of reprogramming strategies to overcome therapeutic resistance in tumors traditionally refractory to immunotherapy. A less explored but equally promising avenue involves Toll-like receptor (TLR) stimulation in Tregs, which can alter their suppressive functionality and metabolic profile. However, the precise molecular mechanisms governing this process, as well as its long-term immunological impact, remain to be fully elucidated in preclinical and clinical settings.

Despite these advances, several key questions remain unanswered. The stability and reversibility of Treg plasticity, the safety of long-term modulation, and the identification of tumor-specific Treg markers remain critical challenges. Furthermore, distinguishing between tissue-suppressive and tissue-repairing Tregs cells is essential to prevent systemic autoimmunity. Addressing these questions will be crucial to achieving Treg cell-targeted strategies that lead to durable and clinically effective cancer immunotherapies.
